# Design and usability evaluation of a mobile application for self-care among Iranian adolescents

**DOI:** 10.1186/s12889-024-18341-z

**Published:** 2024-03-25

**Authors:** Razieh Rezaee, Mohtasham Ghaffari, Reza Rabiei, Amir Kavousi, Sakineh Rakhshanderou

**Affiliations:** 1 Health Education and Health Promotion, School of Public Health and Safety, Shahid Beheshti University of Medical Sciences, Tehran, Iran; 2 Department of Health Information Technology and Management, School of Allied Medical Sciences, Shahid Beheshti University of Medical Sciences, Tehran, Iran; 3 School of Public Health and Safety, Shahid Beheshti University of Medical Sciences, Tehran, Iran; 4 Health Education and Health Promotion, School of Public Health and Safety, Shahid Beheshti University of Medical Sciences, Tabnak Ave., Daneshjou Blvd., Velenjak, Tehran, 19835-35511 Iran

**Keywords:** Self-care, Mobile application, Adolescent Health, mHealth, Mobile phone, Adolescents

## Abstract

**Background:**

Mobile phones can be an ideal platform to engage adolescents to maintain, improve, and promote self-care. Therefore, the current study aims to design and evaluate the usability of a mobile application for self-care in adolescents with a user-centered approach.

**Methods:**

The current applied developmental study was done in four steps. The first step, polling and examining opinions was conducted through in-depth semi-structured interviews, with the aim of user-centered mobile application design with the involvement of 30 participants. The second step, extracting and compiling the educational content related to the main themes of the self-care app, was obtained from national and international guidelines and instructions, including the World Health Organization, the Center for Disease Control and Prevention, the Ministry of Health and Medical Education, etc. In the third step, the initial version of the mobile application was developed. In the fourth step, app usability was evaluated by 30 participants from the target group, 2 weeks after using the app, using the MAUQ questionnaire.

**Results:**

In the first step, 789 codes, 12 sub-categories, and 3 categories were extracted. These codes were used in the design of the mobile application. In the second step, educational information was prepared and arranged in 5 sections (physical activity, nutrition, personal hygiene, risky behaviors and safety and events) in the form of text, images and short videos. In the third step, the mobile application was designed based on step 1 and 2. This application operates in online mode and under the Android operating system. the initial version of the mobile application was developed using JavaScript and Typescript programming languages in a Visual Studio Code environment. In the fourth step, the participants the overall level of usability of the application as very good with an average of 6.28 ± 0.55. The highest average score was given to the user interface and satisfaction with an average score of 6.43 ± 0.58.

**Conclusions:**

The "My-Care" app is a collaboratively designed smartphone app for adolescents that targets 5 dimensions of physical self-care. This app has the potential to teach, assess, and promote self-care among adolescents.

## Introduction

The concept of self-care was first introduced in 1959 by Orem as the "Nursing care deficit theory of self" [1]. Self-care is a conscious, learned, and purposeful practice in which each person uses his acquired abilities and skills in such a way that he can take care of himself personally and independently [2]. As a person grows up, he should learn how to take care of himself from childhood. However, self-care faces unique challenges during adolescence due to developmental factors such as increased risk-taking behaviors [3, 4].

In addition to challenges and problems, it should also be noted that adolescence is a sensitive period, during which adolescents develop their identity, values and beliefs, and behavioral habits, including self-care. Adolescents learn knowledge and skills through experiences to form behaviors related to self-care, which can have a profound effect on a person's development path to adulthood [5]. Many adolescents use mobile applications to monitor and improve their health. Therefore, mobile phones may be an ideal platform for engaging adolescents with interventions to prevent chronic disease risky behaviors [6, 7].

Today, the use of smart mobile phones and tablets to provide healthcare services has expanded. Using practical applications that can be installed on smartphones can be useful and practical to motivate and monitor the treatment and health of people by sending educational messages and audio and video files. Recent technological advances and increased access to smartphones and mHealth among low-income individuals have led to self-care education using mobile-based apps [8–11].

Many people use smartphones as the primary means of accessing healthcare information and managing their health [12]. In addition to instantly accessible healthcare information, mobile health applications improve interactions with the healthcare system [13]. These applications provide accurate and reliable information whenever and wherever users need it, manage users' health, and promote healthy lifestyle 12. Self-care applications have removed the time and place barriers that most healthcare providers face when trying to provide quality care [14].

Thornton et al. (2021) conducted a study aimed at multiple health behavior changes, a self-monitoring mobile application for adolescents: A development and usability study of the Health4Life app. The Health4Life app is a co-designed, self-monitoring smartphone app for adolescents that concurrently targets the Big 6 lifestyle behaviors. Adolescents rated the app as highly acceptable and usable. The app has the potential to efficiently and effectively modify important risk factors for chronic disease among young people [15]. Also, a study by Alves et al. (2021) was conducted to develop and validate mHealth technology to promote self-care for adolescents with diabetes. The results showed that the use of this app by adolescents, considering that it is a very understandable electronic technology, helps them to acquire new knowledge and adhere to healthy practices [16].

As an inevitable part of our daily lives, smartphones are used not only for communication but also for managing a wide range of activities including individual self-care. There have been mobile applications with clinical usage in self-care and self-management of diseases [16, 17]. However, limited research has addressed the capabilities of mobile applications in preventing risky behaviors and self-care of adolescents, and these mainly focused on recommending the features and potential of the applications rather than developing and deploying them [18–20]. The current study aimed to develop a mobile application for self-care of adolescents with no medical condition.

## Materials and methods

The present practical development study was carried out in 4 steps in 2022-2023 as follows:

1. Assessment of users' needs through interviews.
2. Designing and extracting the educational content of the application.
3. Designing and developing the basic structure of the self-care application.
4. Evaluation of the final application.

### Step 1: Determining the functional requirements and features of the application

In the first step, initially, a literature search was conducted to determine the required functions of the application. The literature search was a basis for conducting in-depth semi-structured qualitative interviews. The qualitative interviews were conducted to seek the opinions of adolescents, parents, and specialists about the functional requirements of the application some of which were initially determined using literature search. A heterogeneous sample of adolescents interested in self-care with different needs was selected purposefully. Also, parents or caregivers of eligible adolescents, and health specialists were selected purposefully. Study consent was obtained before the interview. The interviews were conducted by the researcher using interview guide questions. At this step, a total of 30 people (15 students, 10 specialists, and 5 parents) were considered by the research team to check their views.

### Step2: Designing educational content

In the next step, after conducting interviews and discussions and determining the features of the mobile application and educational content, the design of the educational content was done. Given that content quality is one of the most important aspects of health authentic apps, to ensure the quality of health information provided in the mobile application, educational information related to the main themes of the self-care app was obtained from national and international guidelines and instructions.

In this step, educational content related to the main themes of the adolescent self-care mobile application, from national and international guidelines and instructions, including the World Health Organization, the Center for Disease Control and Prevention, the Food and Drug Administration, The Ministry of Health and Medical Education, were obtained [21–24]. The educational content was Prepared and arranged for training and promoting self-care behaviors, considering that we need a valid reference, criterion, and standard for behavior, for this reason, national and international guidelines and instructions were used to prepare this content. These countries include the United States, Australia, and Iran. These guidelines are related to physical activity, nutrition, personal hygiene, high-risk behaviors, and safety and events, and include normal standards and criteria for behaviors related to these 5 dimensions in adolescents. For example, the amount of normal physical activity recommended for adolescents, the importance of eating breakfast and its effects, the importance of sleep and the recommended amount of sleep in adolescents, the harms of using alcoholic beverages, and safety behaviors when dealing with stray animals.

### Step3: Application development

After determining the educational content of the application, the initial version was designed. In this step, operational requirements were extracted and scenarios and Use-Case diagrams were drawn. In line with the design of the user interface of the mobile application, the existing pages and their relationship with each other were modeled in the Figma software. The prototype of the smartphone-based application was designed by specialist designers and experts in software development based on the characteristics and requirements of approved education. To ensure a user-friendly interface, fully considering the needs of the users, the self-care application for adolescents was developed using JavaScript and Typescript programming languages in the Visual Studio Code environment. The JavaScript and Typescript could greatly enhance functionality and usability thanks to JavaScript's versatility in creating dynamic features and handling user interactions. In addition, these languages enable cross-platform compatibility, improved performance through optimized code execution, rapid development iteration, and better scalability and maintainability of your application, ultimately resulting in a more user-friendly and robust mobile app.

We designed the application through conceptual modeling which was composed of three stages: functional, structural, and behavioral. The object-oriented approach was applied for analysis and design. In this study, the validation of the application was checked by the research team to ensure there were no issues with the functionality of the application. The usability testing of the application discussed below helped to ensure the expected functions of the application. Educational content is in the form of videos, related images, and text. This application operates in online mode and under the Android operating system and the user must be online to use educational content and receive messages. In addition, it is possible to receive and download educational videos for users.

### Step4: Application evaluation

At this step, the usability of the application was evaluated through the participation of adolescents, specialists, and parents. The tool used in app evaluation was a questionnaire (MAUQ) developed by Zhou et al. [18] and it is set in three sections: ease of use (5 criteria), user interface and satisfaction (7 criteria), and usefulness (5 criteria) on a 7-point Likert scale ranging from completely disagree (1 point) to completely agree (7 points). This questionnaire was used in previous studies [25–27]. The questionnaire was translated into Persian. Then the questionnaire was translated into English by two experienced English translators to ensure cross-cultural compatibility. The face validity of the questionnaire was evaluated by two medical informatics specialists, as our attempt to involve more participants (five experts were initially considered) failed. The inclusion criteria for the specialists were as follows: having at least five years of experience in their field and being a faculty member at one of the medical universities in Tehran. Based on previously published papers in the field that referred to usability testing of mobile applications for identifying issues with the functionality of applications, we did conduct usability testing for identifying the problems that ultimately could influence the effectiveness and efficiency of the application due to lack of functions. Also after phase 4, it is natural to use the designed app in a real situation, for example in an intervention study, to make sure how the app works. Considering that this study is only a part of the project titled "Design and Evaluation of Mobile Application for Adolescent Self-care" is, the process related to the intervention in the next stage of the project is underway.

The reliability of the questionnaire was re-examined and the internal correlation coefficient of the questions was calculated (Cronbach's alpha = 0.94). Samples participated in the study voluntarily. In this step, the sample of study was composed of 30 participants including students (*N* = 15), specialists (*N* = 10), and parents (*N* = 5) who were recruited by maximum variety view. The participants were asked to install the final version of the self-care application on their mobile phones. After 2 weeks of using the application, the participants were asked to answer the mHealth App Usability Questionnaire (MAUQ). To facilitate reporting the results, we calculated quarters for the total mean value which was 7. The range of mean values for each quarter was as follows: 0–1.75 (poor), 1.76–3.50 (moderate), 3.51–5.25 (good), and 5.26–7 (very good). The inclusion criteria were: Willingness to participate in the study, having an age of 13–15 years for adolescents, informed consent to participate in the study as well as the consent of their parents or legal guardians, and having a smartphone. Necessary explanations about the purpose of the study, and instructions about the app were provided to the participants, and the confidentiality of the collected data was ensured. The collected data were analyzed using SPSS 26 software and by calculating the mean and standard deviation.

## Results

### Findings related to determining the functional requirements and features of the application

The results of the literature search to determine the required functions of the program are reported in Table 1. From the analysis of interviews with 30 people from the target group (Table 2), 789 Initial codes, 12 sub-categories, and 3 categories were extracted, which represent the key features of mobile-based applications and content related to self-care. The codes were merged based on similarity; these codes were then inserted into categories. Attempts were made to have the most homogeneity within the categories and the most heterogeneity between the categories and no data should fall into two categories. The classes included application appearance, application content architecture, and application self-care content (Table 3).

**Table 1:** Results of literature review

Results of literature review related to mobile application	References
➢ There have been no **mobile applications** for healthy adolescent physical self-care	**-**
➢ Self-care-related **mobile applications** were available for the self-care of adolescents with diabetes, cardiovascular disease, asthma, etc	[16, 28–37]
➢ In the **available mobile applications**, they had one or more aspects of self-care	[15, 37–39]
➢ **Mobile applications** that had a combination of images, short videos, text, sound, etc	[15, 40–44]
➢ **Mobile applications** that have games and prizes are more accepted	[45–49]
➢ **Mobile applications** that have used user-centered design	[28, 50]
➢ **Mobile applications** that connect to wearable devices	[31, 51, 52]
➢ The **mobile applications** that were used in the intervention study after the design	[53–55]
**Results of literature review related to self-care in adolescents**	**References**
Studies that have been conducted in different countries in the field of self-care in adolescents: These studies have addressed various aspects of self-care It should be noted that in the present study since the purpose of designing the mobile application was for the physical aspect of self-care of adolescents, dimensions such as psychological, social, etc. were not included	[56–64]

**Table 2:** Demographic characteristics of participants

**Mother's job**	**Father's job**	**Mother's education**	**Father's education**	**Grade**	**Gender**	**Age**	**Students**
Housekeeper	Governmental	Middle school	Bachelor	Seventh	Boy	13	1
Private	Governmental	Bachelor	Bachelor	Seventh	Girl	13	2
Private	Governmental	Bachelor	Bachelor	Ninth	Girl	15	3
Private	Governmental	Bachelor	Bachelor	Eighth	Boy	14	4
Housekeeper	Self-employment	High school	Associate Degree	Eighth	Girl	14	5
Housekeeper	Self-employment	High school	High school	Eighth	Girl	14	6
Housekeeper	Private	High school	Masters	Seventh	Boy	13	7
Private	Governmental	Masters	Bachelor	Ninth	Girl	15	8
Housekeeper	Self-employment	Middle school	Bachelor	Eighth	Boy	14	9
Housekeeper	Private	Middle school	High school	Eighth	Boy	14	10
private	Private	Associate Degree	Associate Degree	Ninth	Boy	15	11
Housekeeper	Self-employment	Elementary school	Middle school	Ninth	Girl	15	12
Housekeeper	Governmental	Middle school	Associate Degree	Seventh	Girl	13	13
Governmental	Governmental	Associate Degree	Bachelor	Ninth	Boy	15	14
Housekeeper	Self-employment	Middle school	High school	Eighth	Girl	14	15
**Job**			**Field of Study**	**Degree of education**	**Gender**	**Age**	**Health Specialists**
Academic Board			Academic Board	Ph.D	Male	47	16
Academic Board			Academic Board	Academic Board	Female	53	17
Academic Jihad			Health education and health promotion	Ph.D	Female	39	18
Academic Board			Medical Informatics	Ph.D	Male	33	19
Academic Jihad			Medical Informatics	Masters	Female	31	20
Academic Board			Health education and health promotion	Ph.D	Female	41	21
School teacher			Public health	Masters	Female	536	22
Nutritionist			Nutrition	Ph.D	Male	38	23
Academic Board			Medical Informatics	Ph.D	Female	29	24
**Nutritionist**			Nutrition	Ph.D	Female	35	25
**Job**				**Degree of education**	**Gender**	**Age**	**Parents**
			Private	Masters	Male	38	26
			Self-employment	Masters	Male	43	27
			Housekeeper	Bachelor	Female	39	28
			Governmental	Bachelor	Male	47	29
			Governmental	Bachelor	Female	40	30

**Table 3:** Codes, categories, and subcategories of key features of mobile applications for adolescent self-care

**An example of extracted codes**	**Sub-categories**	**Categories**
1. The use of images (relevant, appropriate, clear)	Application graphics	**Application appearance**
2. The use of colors suitable for adolescents (relaxing, happy, energizing)		
3. The use of indicator arrow in the required sections for better understanding		
1. Tabular form of the screen	Structuring	
2. Having a different sequence in the sections in order not to cause boredom in the user		
3. Convenient display of content in each section (easy to load)		
1. The use of voice or sound for more impact	Using new media	**The content architecture of the application**
2. The use of films or short videos		
3. The use of animation		
1. Being free	Technical capabilities of the application	
2. Application flexibility based on user preferences		
3. The possibility of step-by-step access to each section		
1. Concise and useful content of each section	Training considerations	
2. Having a conclusion at the end of each section		
3. Non-presentation and teaching inappropriate content		
1. The possibility of adding a spiritual part to the application	Suggested add-on capabilities	
2. The possibility of adding a psycho section in the application		
3. The possibility of continuous follow-up of the user's health		
1. Having an interactive function	General requirements of the application	
2. Being user-friendly		
3. Having a public aspect		
1. Emphasizing the importance of breakfast	Nutrition	**Self-care application content**
2. Providing a food plan to reduce stress		
3. Providing a food plan to strengthen memory		
1. Teaching sports movements	Physical activity	
2. Using arrows to show movements related to sports		
3. Determining suitable times for exercise		
1. Women's health education	Personal hygiene	
2. The possibility of dental consultation		
3. Sleep hygiene education		
1. Creating a website for the risky behaviors section	Risky behaviors	
2. Teaching methods for preventing risky behaviors		
3. Teaching strategies to deal with risky behaviors		
1. Providing solutions to deal with incidents	Events	
2. Increasing awareness about the consequences of injuries and events		
3. Teaching methods of preventing events and injuries		

## Findings related to designing educational content

At this stage, the educational information related to the main themes of the adolescent self-care mobile application was obtained from national and international guidelines and instructions, including the World Health Organization, the Center for Disease Control and Prevention, food and Drug Administration, The Ministry of Health and Medical Education, were obtained [21–24]. This information was prepared and arranged into 5 sections, including physical activity, nutrition, personal hygiene, risky behaviors, and safety and events. In (Table 4) the educational content of each section is given. This information was prepared and arranged into 5 sections, including physical activity, nutrition, personal hygiene, risky behaviors, and safety and events.

**Table 4:** The educational content of the application sections

**Sections**	**Educational content**
***Physical activity***	Advantages and disadvantages of physical activity, the amount of physical activity for adolescents, options for doing physical activity, steps to do a physical activity session, how to start physical activity, important points in doing physical activity, and how to make physical activity attractive
***Nutrition***	The importance of healthy food for adolescents, types of healthy food for adolescents, amount of food consumption, breakfast and its importance, types of eating habits, and vitamins and minerals
***Personal hygiene***	Body hygiene, hand hygiene, oral and dental hygiene, skin and hair hygiene, and sleep hygiene
***Risky behaviors***	Risky behaviors in adolescents, drugs and alcoholic beverages, smoking and hookah, suicide, violence, and sexual relations
***Safety and events***	Traffic accidents, domestic events or events at home, events in sports environments, and events in public environments

## Findings related to Application development

**Fig. 1:**
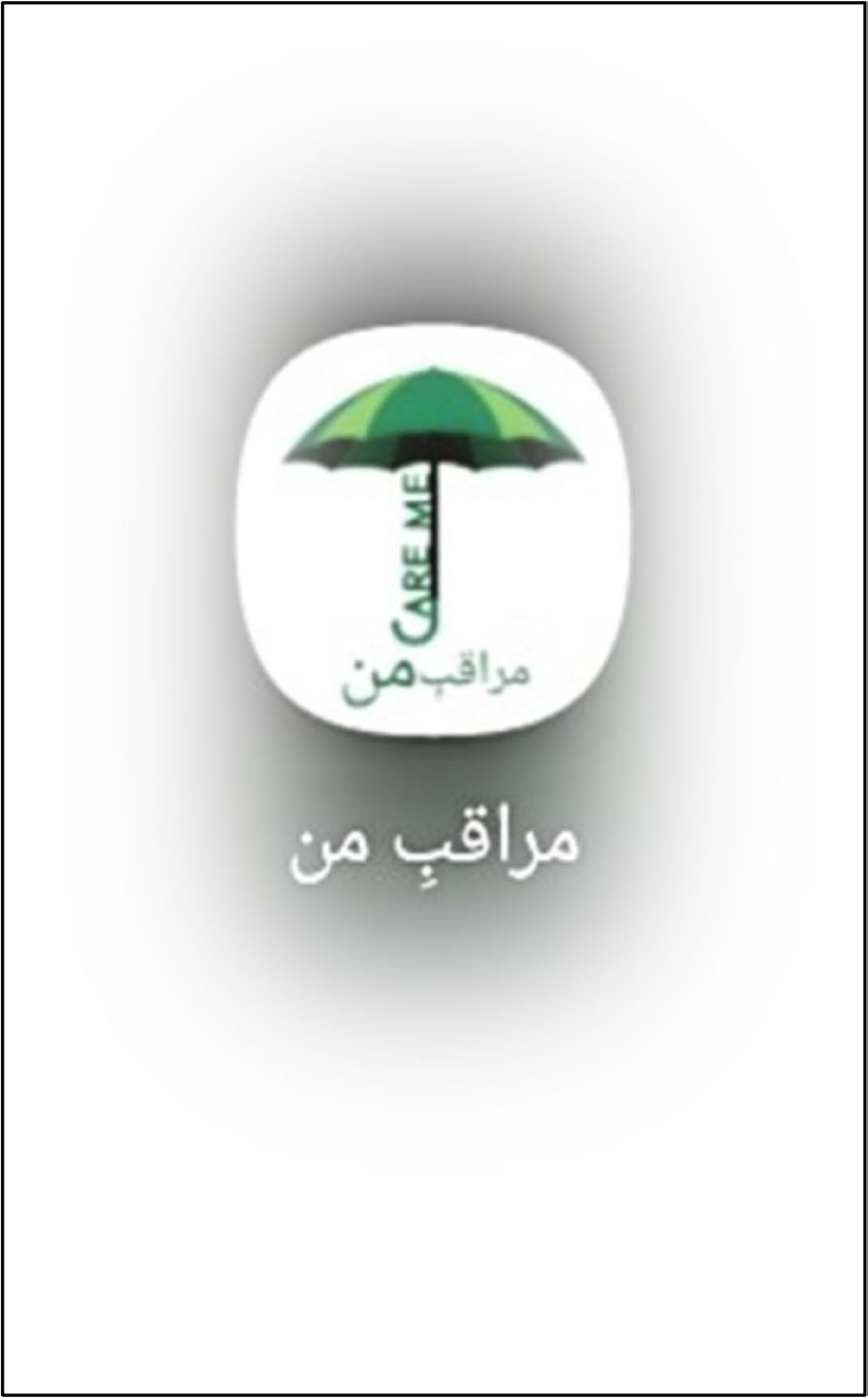
"Logo" of the Care Me application

**Fig. 2:**
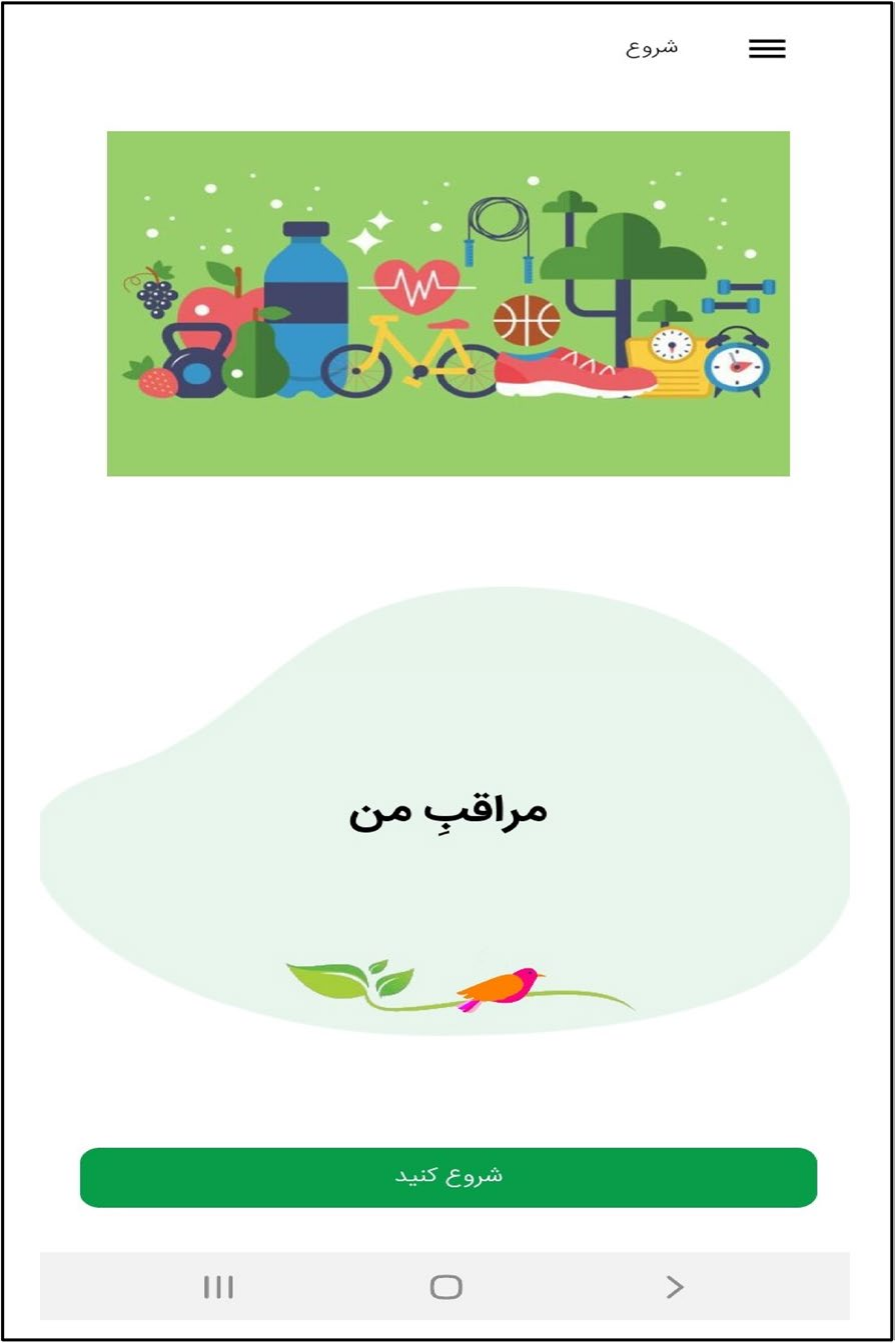
The "Start page" section in the application

**Fig. 3:**
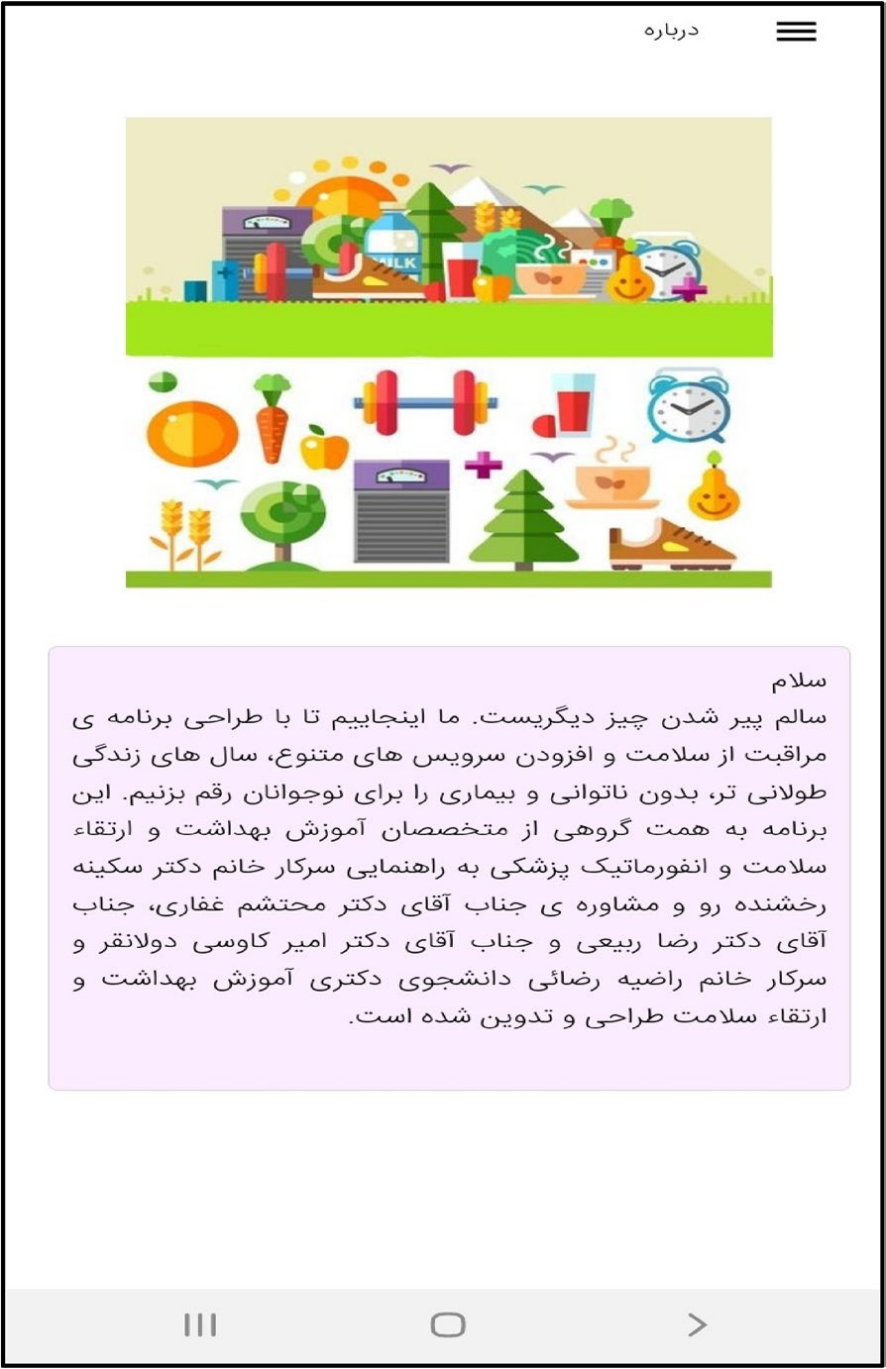
The "About Us" section in the application

**Fig. 4:**
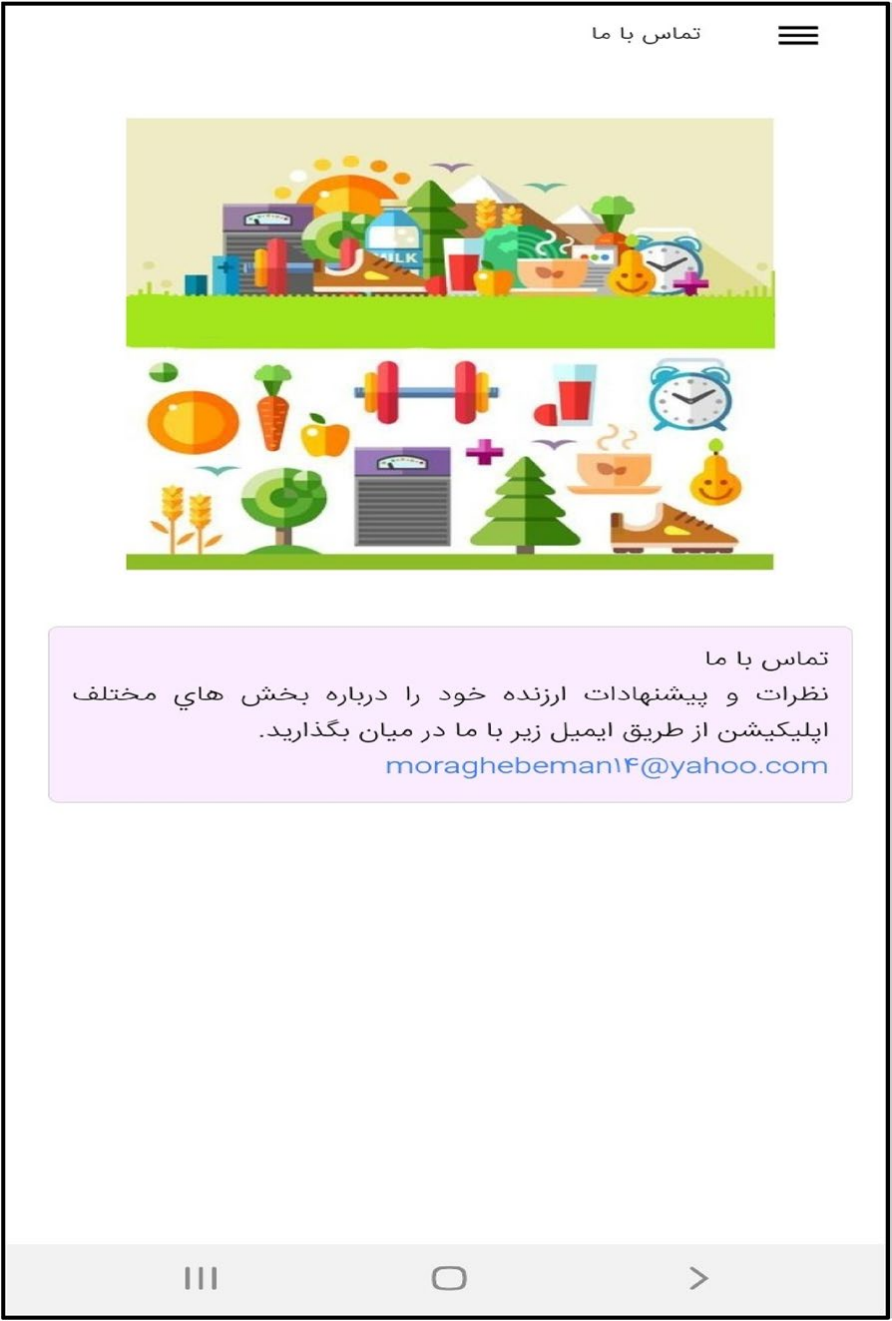
The "Contact Us" section in the application

**Fig. 5:**
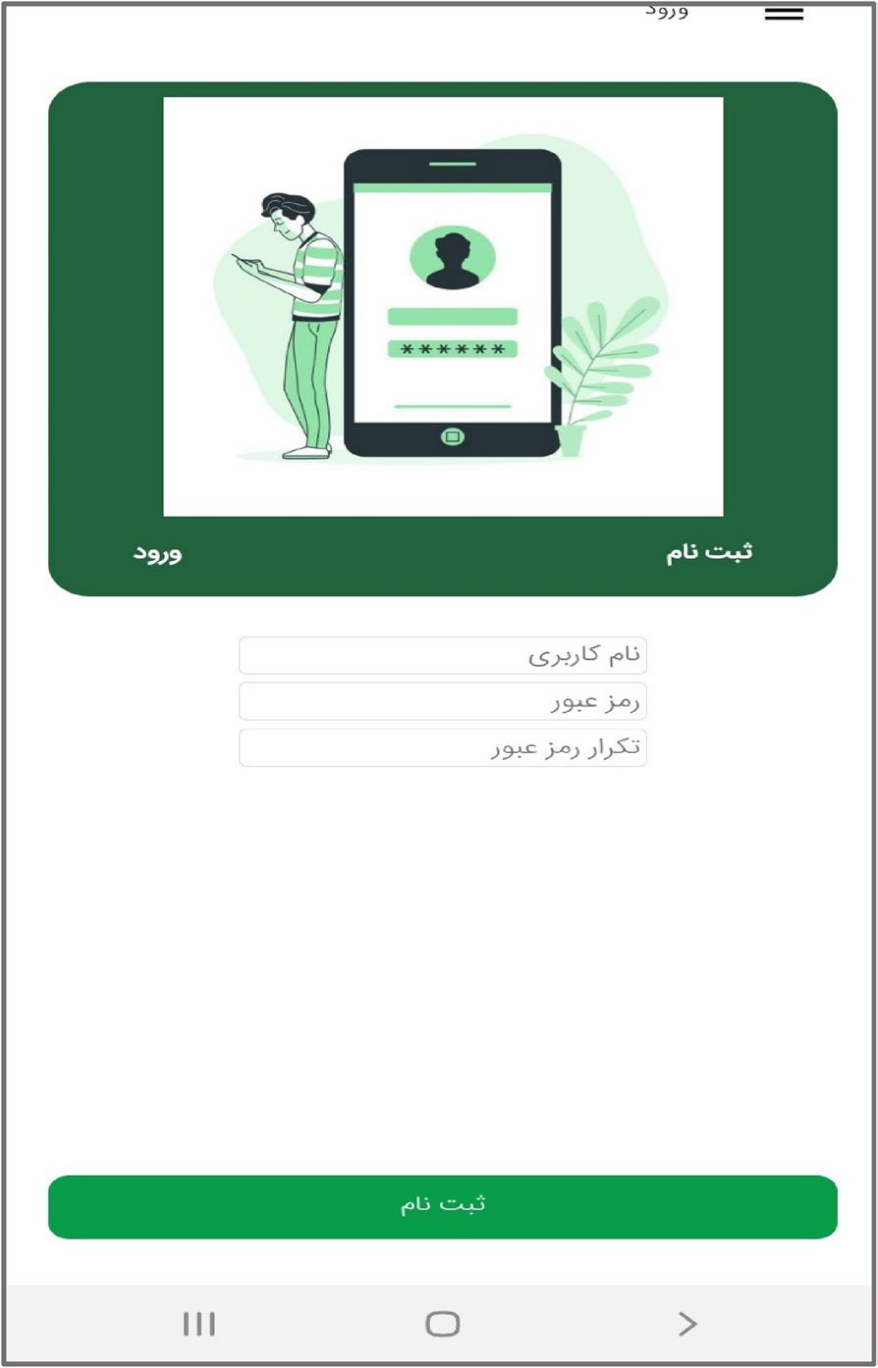
"Login" section in the application

**Fig. 6:**
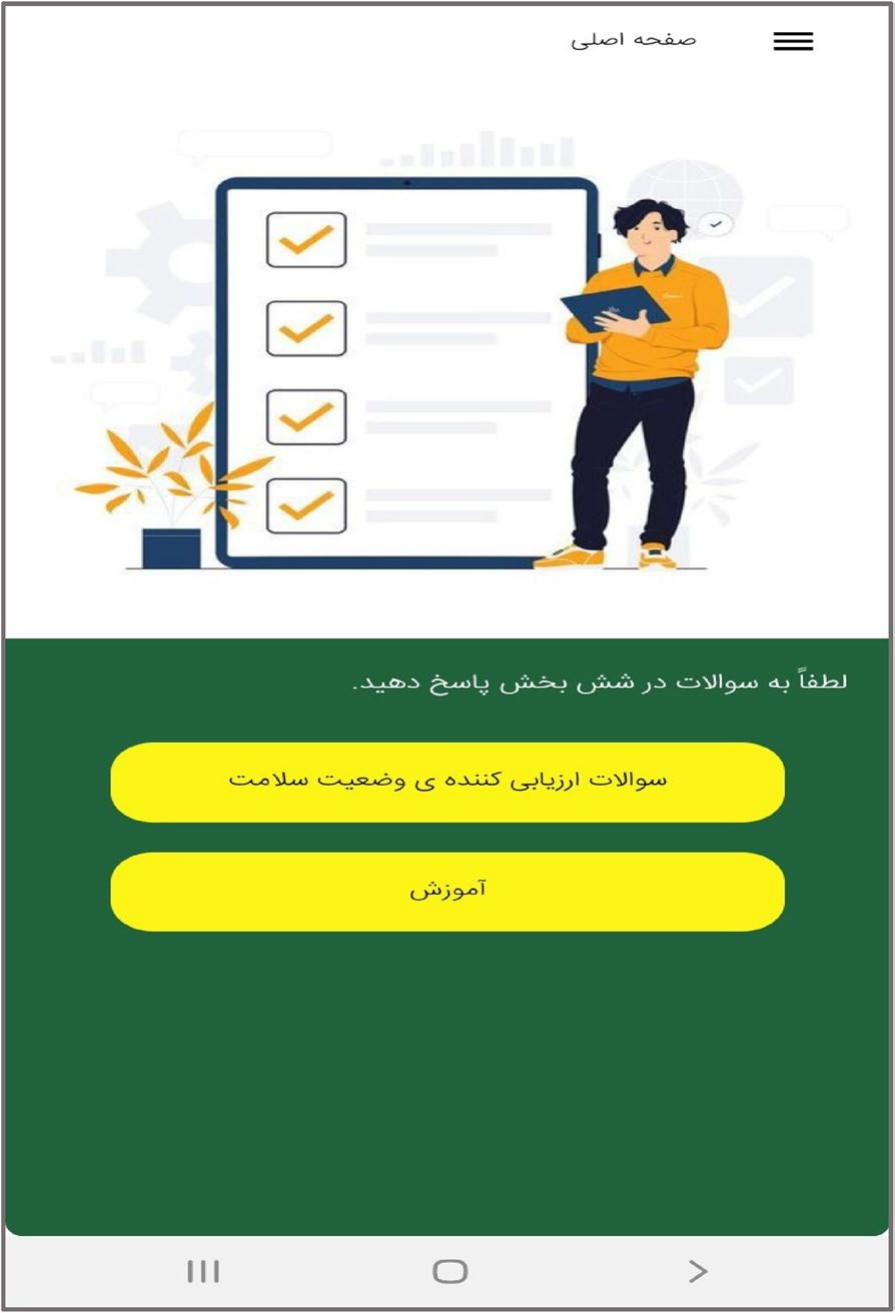
"Main page" section in the application

**Fig. 7:**
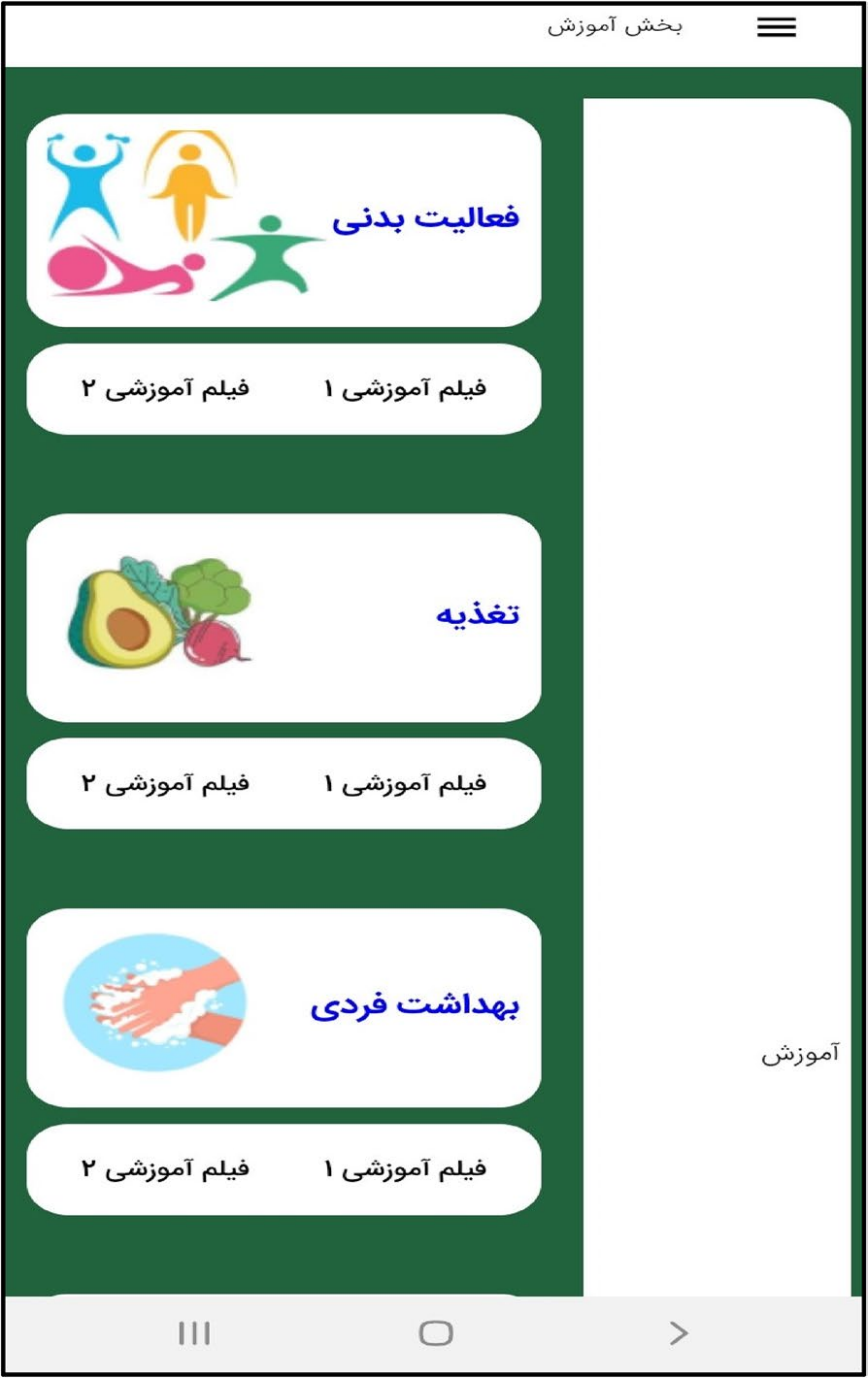
The "Training" section in the application

**Fig. 8:**
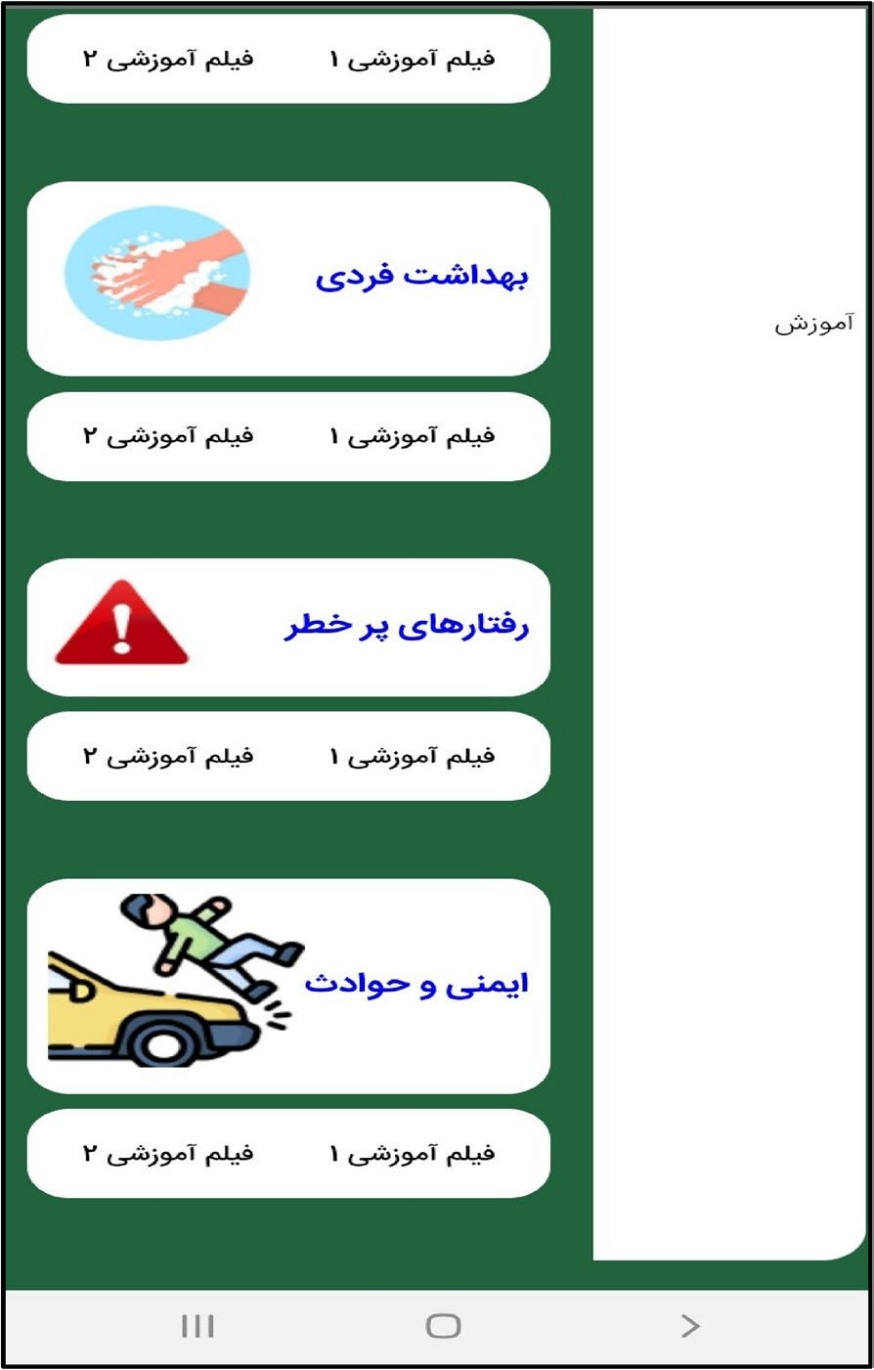
The "Training" section in the application

**Fig. 9:**
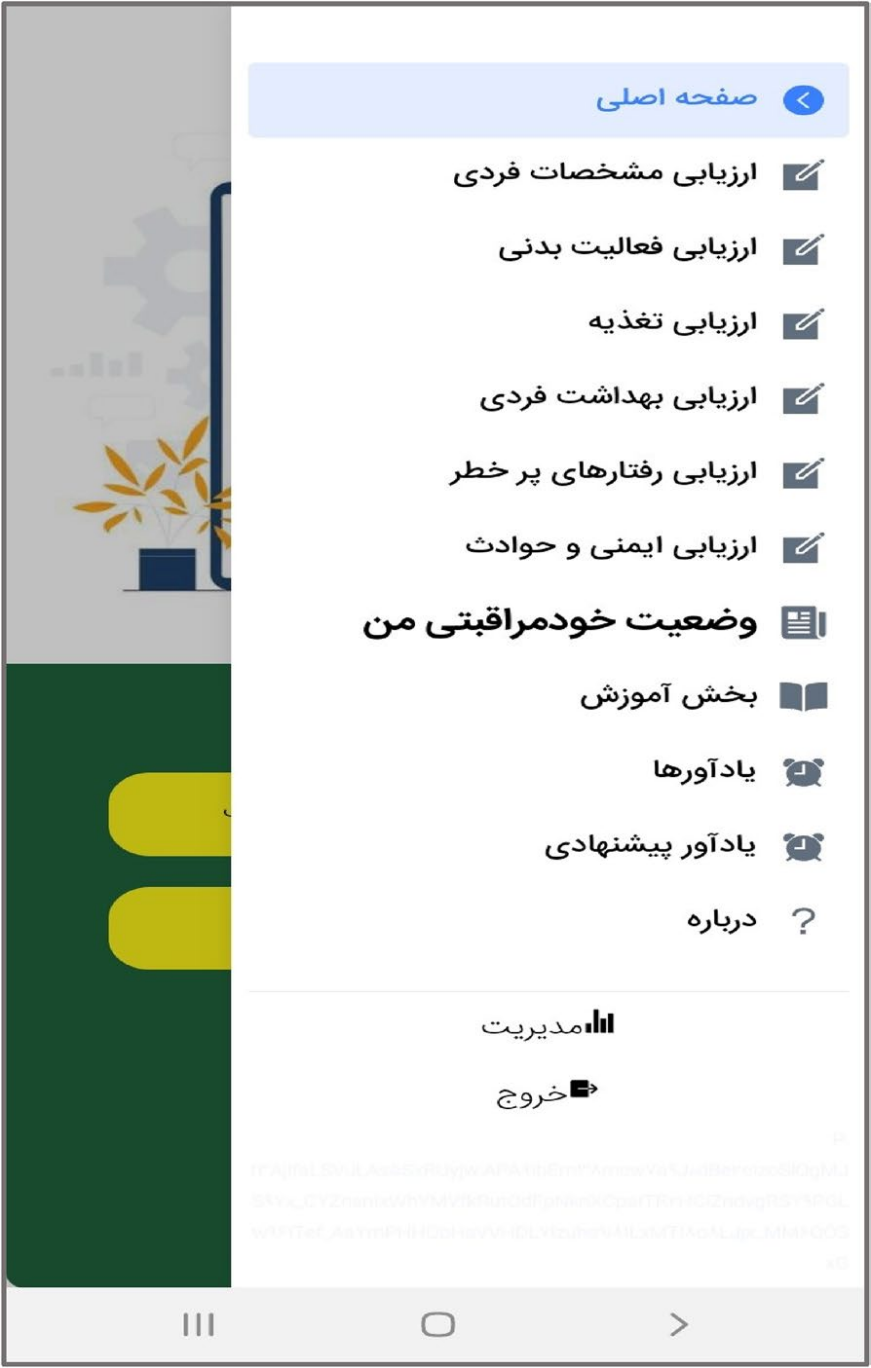
"Menu" section in the application

**Fig. 10:**
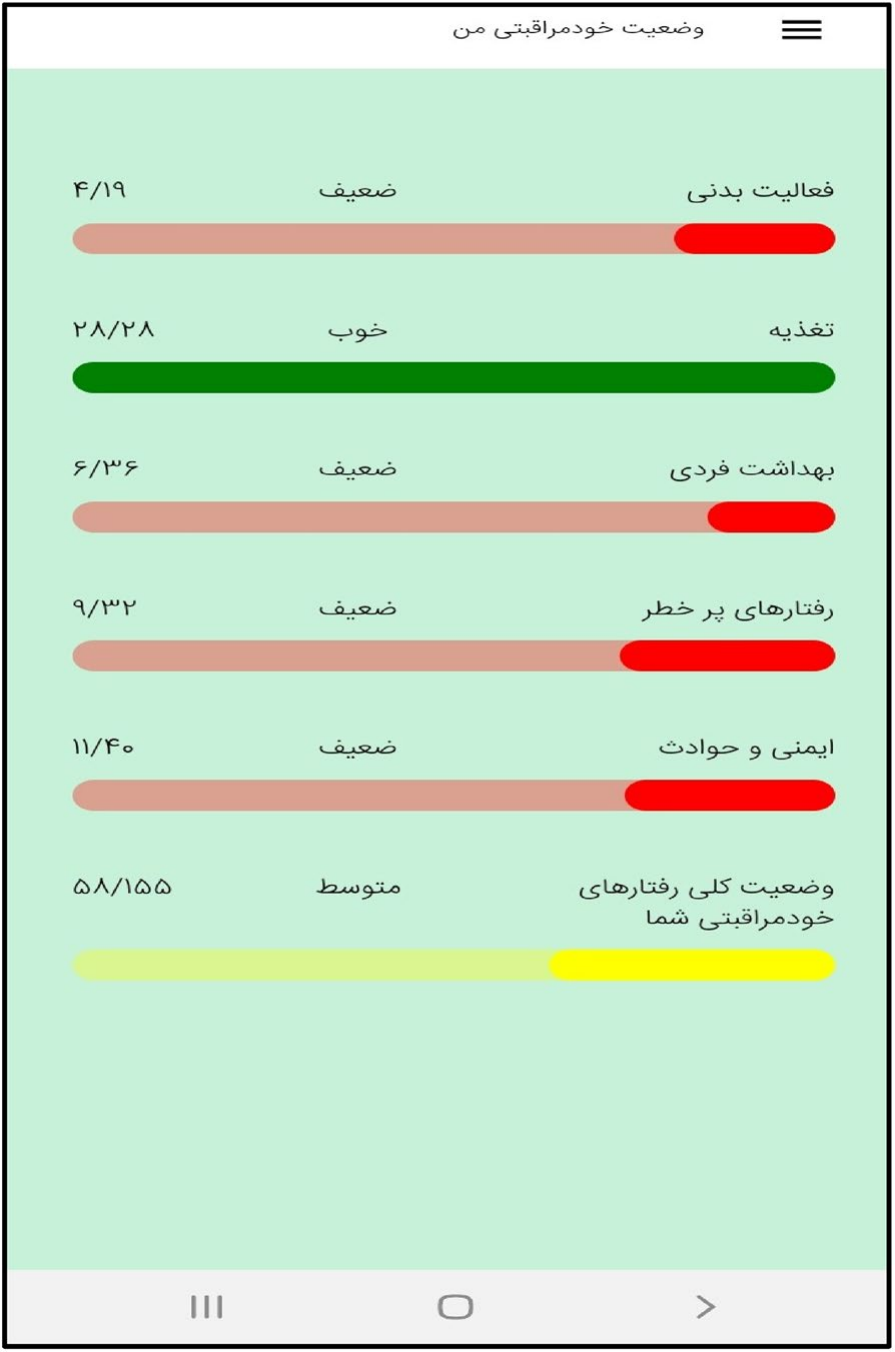
"My self-care status" in the application

**Fig.11:**
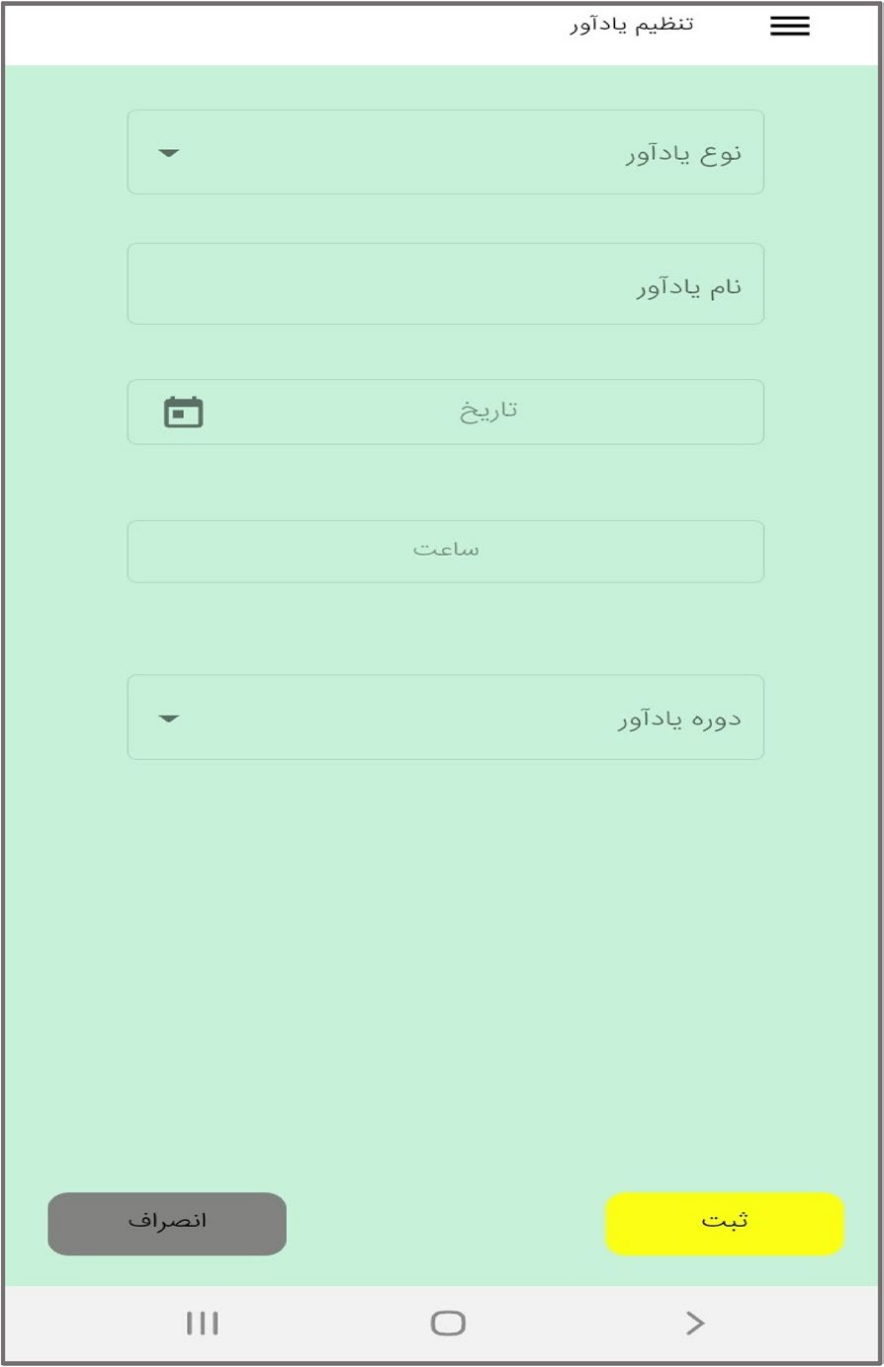
"Reminder setting "section in the application

**Fig. 12:**
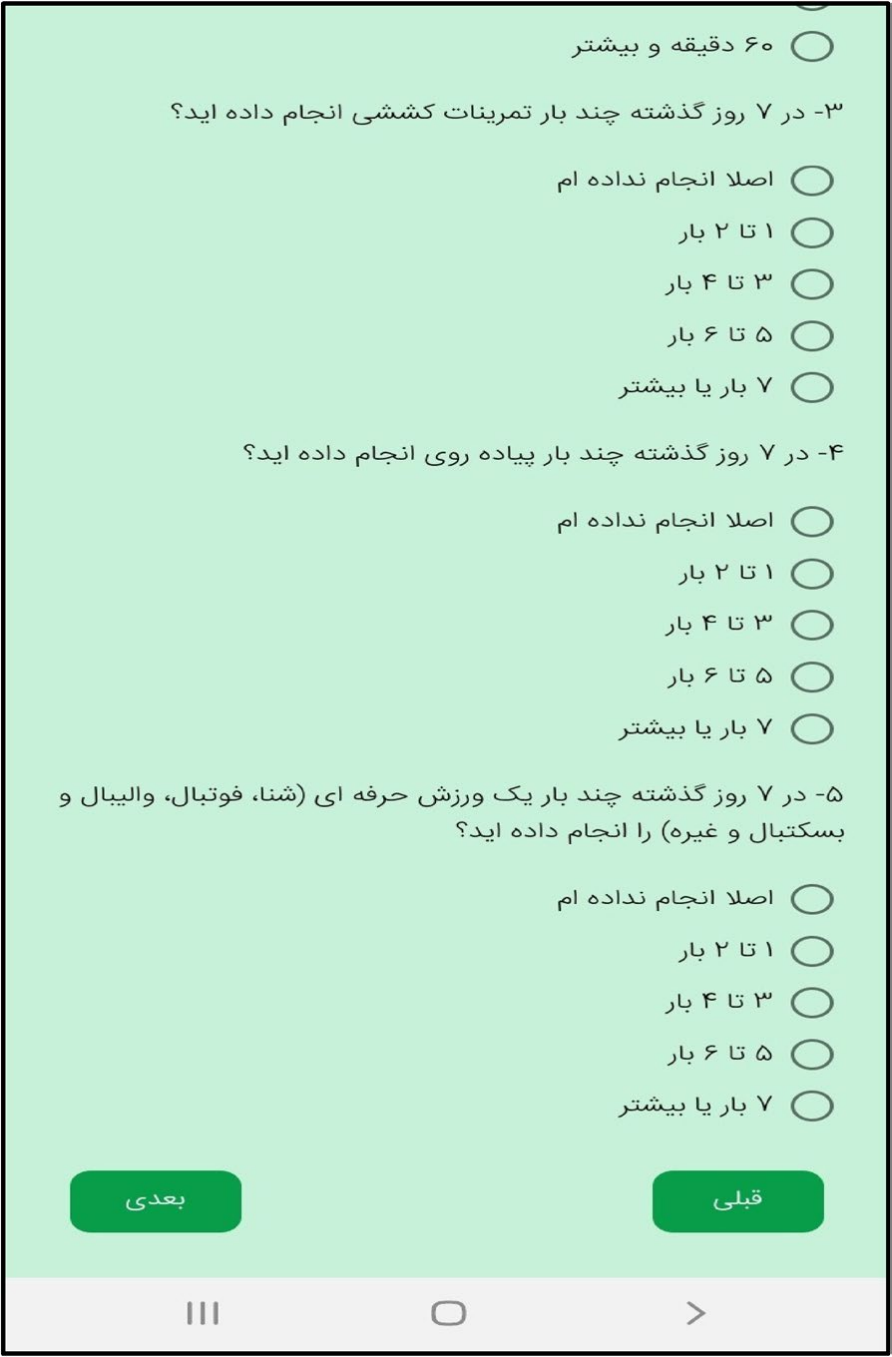
"Questions page" of the Care Me application

In the third step, the mobile application " Care Me " was designed based on the results obtained in the previous stages. conceptual modeling of the app included operational, structural, and behavioral modeling that was created through object-oriented analysis. To develop a scalable application with evolving data schemas, Mongo DB was used. Angular as an open-source JavaScript framework was employed to design the user interface. NodeJs was also utilized to create the server side of our application. The main app logo is shown in Fig. 1. At the beginning of the app, start page, the app introduction, contact us, and the login page are displayed (Figs. 2, 3, 4, 5). The main services of the app include training (Figs. 6, 7, 8), assessing my health status (Fig. 9), my self-care status (Figs. 9, 10), setting reminders (Fig. 11), questions page (Fig. 12) and sending encouraging messages First, to use the mobile application, adolescents must "register" and enter the main page of the application. In this section, adolescents must answer "health status assessment questions" in 6 sections (demographic characteristics, physical activity, nutrition, personal hygiene, risky behaviors, and safety and events). After answering the questions in the "My self-care status" section, adolescents can evaluate the results of their self-care status in 5 sections (physical activity, nutrition, personal hygiene, risky behaviors, and safety and events) and their general self-care status in one of Observe 3 statuses (good, medium and weak). If the person's self-care status is in one of the two states of " medium " and "weak", healthy and encouraging messages will be displayed every week to create a "good" self-care status for people in the form of notifications. In the "Education" section, there is educational content along with pictures and short videos to convey health and self-care concepts to improve physical activity, nutrition, and personal health, reduce risky behaviors improve safety, and prevent events. Also, the "reminder setting" section is provided to users to record and save their health behaviors in this section to remind or warn them at the desired time and not to forget.

## Findings related to application evaluation

Table 5 shows the characteristics of the participants who participated in the usability evaluation test. The information obtained from the questionnaires was entered into the SPSS 16 software by the researcher and analyzed using descriptive statistics (frequency and percentage). Thirty participants entered in the application evaluation. Participants, overall in 3 sub-groups, were at least 13 years old and at most 56 years old. The level of education of the participants varied from seventh grade to Ph.D., and the Ph.D. degree was the highest frequency.

**Table 5:** Characteristics of participants in usability evaluation

**Participants**	**Variables**	**N (%)**
**Students**	Age range (13–15)	**15 (50)**
	Gender (7 Girl- 8 Boy)	
	Education level (5 (7th)- 5 (8th)- 5 (9th))	
**Specialists**	Age range (33–56)	**10 (33.33)**
	Gender (5 Female- 5 Male)	
	Education level (Ph.D.)	
**Parents**	Age range (36–50)	**5 (16.67)**
	Gender (3 Female- 2 Male)	
	Education level (1 Diploma, 3 Bachelor, 1 M.Sc.)	

After the data analysis using descriptive statistics (mean and standard deviation), the results of usability evaluation were prepared in Table 6. The average score of the applicability of the app was 6.28 ± 0.55. Among the usability criteria, the highest average score was given to the user interface and satisfaction with an average score of 6.43 ± 0.58. The average score of 6.32 ± 0.51 was obtained for the ease of use of the application, and then the average score of 6.02 ± 0.78 was obtained for the usefulness of the application. As score range of 5.26—7 is considered very good, findings showed that all dimensions of app, usability as well as total evaluation of app usability were in very good levels.

**Table 6:** Descriptive statistics of the mHealth usability questionnaire main criteria (*n* = 30) Disagreement (1–3) Borderline (4) Agreement (5–7)

**Dimensions of Usability**	**Questions**	**Disagreement**	**Borderline**	**Agreement**	**Mean ± SD**	**Level**
		**N (%)**	**N (%)**	**N (%)**		
***Ease of Use***	Q1: Easy to use	-	-	30(100)	6.32 ± .51	Very Good
	Q2: Ease of learning	-	-	30(100)		
	Q3: Smooth navigation	-	1(3.3)	29(96.7)		
	Q4: Use all features	-	-	30(100)		
	Q5: Correction of mistakes	-	1(3.3)	29(96.7)		
***Interface and Satisfaction***	Q6: Interest	-	2(6.7)	28(93.3)	6.43 ± .58	Very Good
	Q7: Information organization	-	1(3.3)	29(96.7)		
	Q8: Notification of progress	-	-	30(100)		
	Q9: Comfort in the environment	-	1(3.3)	29(96.7)		
	Q10: Time spent using system	-	-	30(100)		
	Q11: Reuse	-	1(3.3)	29(96.7)		
	Q12: Satisfaction	-	1(3.3)	29(96.7)		
***Usefulness***	Q13: Usefulness	-	3(10)	27(90)	6.43 ± .58	Very Good
	Q14: Access to services	3(10)	6(20)	21(70)		
	Q15: Effective management	1(3.3)	1(3.3)	28(93.3)		
	Q16: Availability of function	-	2(6.7)	28(93.3)		
	Q17: Facilitating access to services	1(3.3)	1(3.3)	28(93.3)		
***Total***					6.28 ± .55	Very Good

## Discussion

Based on the results, as much as possible, we used the extracted codes from the interviews in the design of the app and its educational content. For example, in the educational content of the application, we considered the extracted codes in creating clear images, short videos, user-friendly feature, general aspect, and brief and useful information about the importance of breakfast, exercise training, sleep hygiene, prevention of risky behaviors and dealing with events.

Adolescence is a certain time for preventive interventions such as self-care behaviors [65]. Mobile Health Applications (MHAs) are becoming progressively common as digital interventions in a wide range of health-related applications in most healthcare sectors [66]. Smartphone applications can be considered as a protector of healthcare programs because anyone can use them in a completely individual way. They have the opportunity to collect information in real-time provide graphical demonstrations and also interchange and interact with the information [67]. As for the use of mobile applications, most adolescents are skilled at these technologies. Similarly, continual reports indicate that utilizing modern mobile electronic technologies in health interventions for young people might be a suitable strategy to address self, shared, or joint care in a manner that is resource-efficient [68–70].

In the present study, a mobile application for self-care of adolescents was designed, which includes 5 sections of physical activity, nutrition, personal hygiene, risky behaviors and safety, and events with educational content. Similarly, in the study conducted by Thornton et al., an app with 6 sections was designed, 5 of which include physical activity, nutrition, smoking and alcohol consumption, and sleep hygiene, which are consistent with the sections of the present study [15]. In the study of Frontini et al. and Likhitweerawong et al., the existence of two parts of physical activity and nutrition in the mobile application is similar to the present study [37, 38]. Also, in the study of Jeong et al., the existence of an educational section related to suicide in adolescents in the mobile application is similar to our study [39]. Health apps that focus on specific dimensions of health, such as the app of the present study, which was designed in the field of the physical dimension, may have an advantage because they can more comprehensively deal with issues related to that dimension although a more definite claim in this field requires other researches.

Rivera et al. used the user-centered design method in the design of the mobile application in their study. The user-centered design of this study is completely similar to the user-centered design of the mobile application of the current study [28]. In this way, in this research, interviews were used to get users' opinions, and interviews were conducted with adolescents, parents, and experts. Similarly, in the study of Birnie et al., a user-centered approach was used in the design of a mobile application, and children, adolescents, and parents participated in semi-structured individual interviews, and experts participated in focus groups [50]. User-centered design, a philosophy that prioritizes users' needs and preferences, is critical to creating successful applications.

In this mobile application, educational content about self-care was presented in 5 sections in the form of educational text, short film, picture, motivational messages, and reminder messages. In the study of Nielsen et al., quizzes; games; self-refection; Challenges; Stories by Peers (stories from peers and information from a doctor); condom tips, pep talks, and boosting; and Random Facts were included in the program based on the inputs of adolescents [40]. In the present study, games, and stories were not used, unlike the study of Nielsen et al. but there are educational content and test in the two studies. These apps were made to change the behavior of adolescents to self-care, therefore the educational content should be attractive and entertaining and multimedia.

Also, in Thornton et al.'s study, the methods of providing notification and feedback on behavior, self-monitoring and goal setting, and providing inspirational quotes, images, symbols, or fonts were used [15]. Similarly, there is self-monitoring and providing feedback and messages, progress, a dashboard, and a menu in the current study mobile application, but unlike Thornton et al.'s study, the diary section and rewards are not designed in the current study app. In the current research, music without words was used on the opening page of the program, which can be turned off and on. Similarly, in the study of Alves et al. as a stress-coping tool, the application brings a playlist with instrumental songs to promote relaxation [16].

In the present study, the participants who attended the usability test rated the overall level of usability of the mobile application as "very good". In Thornton et al.'s study, in the usability evaluation test, adolescents evaluated the Health4Life app as very acceptable and usable [15]. In Alves et al.'s study, the Smartphone Usability questionnaire was employed for technical validation. The content validation process was mediated by the Suitability Assessment of Materials tool, characterizing the APP as “Superior Material” We concluded, therefore, that the use of this material by adolescents will favor the acquisition of new knowledge and adherence to healthy practices, considering that it is a highly intelligible electronic technology [16]. The results of these two studies are similar to the present study.

Since the challenges and problems related to the app were identified and corrected several times by the research team during the design, installation and implementation process, which is confirmed by the findings related to the usability score and the very good level of the designed app. there was no any need to change after the finalization of the app and the usability testing step. Of course, perhaps by using this app in other studies and populations, further modifications may be unavoidable.

Considering that maintaining privacy and the existence of a password in the app is essential to gain the confidence and trust of users. In this study and other studies, they have mentioned the existence of a password in the app [15, 71, 72]. This was one of the features mentioned by users in the design of the app. This helps the user to confidently enter his information and use the app.

In the present study, an attempt was made to develop an app that included most of the features of another similar app in a single app to be able to meet users' requirements, One of the most important features of an ideal app to compete with other apps is to comply with the principles of usability. It is expected that adolescents will improve their health information by using such technologies, and by improving their beliefs and attitudes related to health, they will adopt appropriate self-care behaviors to achieve the desired health status.

In relation to potential future directions for the application, further user testing, updates, or adaptations based on ongoing user feedback has been planned to improve the usability of the application, along with collaborations with Iranian Ministry of Health and Medical Education and Iranian Ministry of Education for broader impact. In addition, the application has the flexibility to be customized based on diverse cultural contexts in different regions. For example, the main features and functions of the application including educational content, self-assessment, and setting reminders could be developed or customized considering cultural contexts.

## Strengths and limitations

From the positive aspects, the following can be mentioned:

1. Positive feedback from users about being aware of their health status.
2. Positive feedback about the possibility of setting reminders and notifications for their behaviors.
3. Stating that they haven't had a complete self-care app so far and this app gives good information about their health.
4. Also, another aspect is the possibility of receiving user data output for the admin.

Two formerly mentioned aspects besides setting an audience contact way (e.g. email) in Care Me app. could be considered as features of interactivity for such applications.

We had some barriers and challenges during the collaborative design process that were addressed and resolved as following;

1. Initially, the mobile application was not running on the lower Android operating system, which was solved by the programming team.
2. Another issue was related to the principles of working with the app, which required more explanations for some users. This was partially solved by adding a description page at the beginning of the app.
3. Due to the use of VPN by some users, entering the app was facing a problem, which required disconnecting the VPN to use the app. This problem was solved by disconnecting the VPN for users who were facing the problem of not opening the app.
4. Also, to enter the app, it was necessary to register users, otherwise it was not possible to log in. Also, the process of registering and defining a password was a bit difficult because users needed to define a strong 6-digit password. This was partially solved by adding a description page at the beginning of the app.

Each study has own limitations. When installing and using the app by users, there were the following problems:

1. One of the limitations was that users had to go online to use the app and receive motivational and reminder messages. The possibility of running the application an offline mode could help to improve its usage.
2. Among the limitations of this research, can be mentioned that due to the lack of financial support, the design and development of more sections and the use of some animations and educational videos were more limited.
3. Because of limited time and resources, coding and grouping of qualitative data was completed by only one researcher.
4. The qualitative feedback is based on a small number of adolescents, parents, and experts generalizing their views to a wider population and should be exercised with caution.

## Conclusion

Considering the ever-increasing expansion of mobile applications and the increase of their users, especially in the age group of adolescents, investing in these technologies in the field of health is obvious. On the other hand, considering that investing in health in adolescents is effective in health and prevention of diseases in adulthood, it is necessary to design applications to maintain and improve health in adolescents by specialists. Based on this, the mobile application " Care Me " was designed and evaluated with the aim of improving self-care in adolescents. The participatory approach used for designing the application helped to consider five main dimensions of physical self-care with practical implications for schools and relevant health centers. The approaches used for designing and developing the application in the current study could provide a basis for developing other mobile applications for adolescents’ self-care. It is expected that by adopting these types of apps in relevant interventions, favorable health outcomes, including improved self-care behaviors of adolescents could be achieved. The effect of this mobile application on the self-care status of adolescents will be evaluated in a randomized controlled trial in Iran (IR.SBMU.PHNS.REC.1400.073) and will be reported separately. The mobile application developed in this study could help to assess the short term effects as well as the long term outcomes in adolescent’s self-care behaviors.

## Data Availability

Materials and data are available upon reasonable request from the corresponding author by e-mail at s_rakhshanderou@sbmu.ac.ir.

## Abbreviations

MAUQ: MHealth App Usability Questionnaire
MHAs: Mobile Health Applications
MongoDB: The Developer Data Platform
Node.js: Open-source, cross-platform JavaScript runtime environment and library
JavaScript: Programming language and core technology of the World Wide Web
